# Differential impacts of digital interactive media and print media on mental health: mediating mechanism of health literacy and group heterogeneity

**DOI:** 10.3389/fpubh.2025.1618570

**Published:** 2025-09-04

**Authors:** Junfeng Yuan, Lin Luo

**Affiliations:** ^1^School of Physical Education, Guizhou Normal University, Guiyang, China; ^2^Key Laboratory of Brain Function and Brain Disease Prevention and Treatment of Guizhou Province, Guiyang, China

**Keywords:** print media, digital interactive media, health literacy, depression, anxiety

## Abstract

**Background:**

In the digital era, media have become critical conduits for information dissemination, exerting increasing influence on mental health. Traditional print media and digital interactive media differ significantly in how individuals access and process information, potentially influencing psychological well-being through distinct mechanisms. Health literacy—the integrated capacity to acquire, understand, and apply health information—has been recognized as a key mediator in the relationship between media use and mental health. However, the precise mechanisms and age-related variations of this mediating effect remain underexplored.

**Objective:**

This study aims to investigate the differential pathways through which print media and digital interactive media influence depressive symptoms (measured by the Patient Health Questionnaire, PHQ) and anxiety symptoms (measured by the Generalized Anxiety Disorder scale, GAD), with a focus on the mediating role of health literacy and its heterogeneity across age groups.

**Methods:**

Using data from the 2021 China Mental and Behavioral Survey, a total of 9,966 adults aged 19 and older were selected as the study sample. Key variables included: independent variables—frequency of print media and digital interactive media use; mediating variables—three dimensions of health literacy (healthcare, disease prevention, and health promotion); and dependent variables—scores on depression and anxiety symptoms. Descriptive statistics, Spearman correlation analysis, ANOVA, and bootstrap-based mediation tests (resampled 1,000 times) were conducted to systematically examine how different media types affect mental health through specific mechanisms.

**Results:**

Digital interactive media use was significantly positively correlated with all dimensions of health literacy (*r* = 0.242–0.297, *p* < 0.01). It directly reduced levels of depression and anxiety (PHQ-9 effect size = −0.138; GAD-7 effect size = −0.145) and partially mediated this effect through the “disease prevention” dimension of health literacy (*β* = −0.021, 95% CI [−0.105, −0.025]). In contrast, print media use was positively associated with depression and anxiety scores (*r* = 0.025–0.039, *p* < 0.05), and all three dimensions of health literacy—"healthcare,” “disease prevention,” and “health promotion”—exerted a suppressing effect on this relationship (*β* = 0.003–0.004). Moreover, these mechanisms varied significantly across age groups: among individuals aged 19–40 and 41–60, the “disease prevention” dimension served as the primary mediator; whereas in the 61 + age group, the “health promotion” dimension emerged as the dominant pathway.

**Conclusion:**

Print and digital interactive media influence mental health through different mechanisms, which vary significantly across age groups. Based on these findings, a stratified intervention strategy—"digital prevention + print promotion”—is recommended: for younger populations, leveraging digital media to enhance disease prevention awareness is essential, while for older populations, an integrated media ecosystem should be developed to reduce cognitive load. This study proposes a media-type-centered framework for mental health intervention, and future research should employ longitudinal designs to establish causal inferences.

## 1 Introduction

Mental health disorders have emerged as a critical global public health issue, posing significant threats to individual well-being and national socioeconomic stability. According to the World Health Organization, approximately 3.8% of the global population suffers from depressive disorders, and 4.1% from anxiety disorders (1, 2). In China, over 100 million people are affected by depression and anxiety, placing a sustained and substantial burden on the national public health system (3). As key vehicles for information dissemination and social engagement in contemporary society, media play an increasingly influential role in shaping mental health outcomes.

### 1.1 The general relationship between media use and mental health

Media use is an integral part of daily life in modern society, exerting complex and multidimensional effects on mental health. According to the “medium is the message” theory, different types of media not only vary in how they present information but also differ significantly in their capacity to evoke and reshape cognitive and sensory experiences (4, 5). Media displacement theory and time displacement hypothesis suggest that increased engagement with digital media often comes at the expense of print media consumption, which may lead to offsetting or even opposing effects on mental health (6, 7).

Existing studies indicate a complex bidirectional relationship between media use and mental health. On the one hand, media can improve mental well-being by providing social support, health information, and mental health resources (8, 9). On the other hand, excessive or inappropriate media use may exacerbate mental health problems (10, 11). However, most of these studies focus on the effects of a single type of media, lacking systematic analyses comparing the differential impacts of various media types (12, 13).

### 1.2 Digital interactive media use and mental health

Digital interactive media encompass diverse forms, including social media platforms (e.g., WeChat, Weibo), online health applications, internet health information portals, and dedicated digital health platforms. These media vary in both effects and reach: social media primarily foster social interaction and emotional expression (14, 15), health apps provide personalized health management tools (16, 17), and professional health platforms offer authoritative medical information and services (18, 19).

A substantial body of empirical evidence supports the positive effects of digital interactive media on mental health. A longitudinal study of retired older adults in the United States found that regular internet use significantly reduced their risk of depression (20, 21). Digital media enhance knowledge acquisition through unique interactive mechanisms: by overcoming temporal and spatial constraints, they lower barriers to accessing health information, enabling users to seek tailored content and support proactively (22, 23). Moreover, digital platforms offer innovative tools for mental healthcare, such as online cognitive behavioral therapy (CBT) and mobile health apps, greatly improving accessibility to mental health services (24, 25).

The “unique mechanisms” of digital interactive media are manifested in three main aspects: (1) real-time interactivity—users receive immediate feedback and support, fostering a sense of social connection and belonging (26, 27); (2) personalized customization—content tailored to user behavior and preferences enhances information relevance and effectiveness (28, 29); (3) social network effects—strengthened social support through connectedness helps reduce loneliness and social isolation (30). These features give digital interactive media unparalleled advantages in promoting mental health.

### 1.3 Print media use and mental health

In contrast, print media mainly include traditional publications such as newspapers and magazines, characterized by linear and static information presentation (31, 32). The relationship between traditional print media and mental health exhibits a more complex pattern. On one hand, print media provide an in-depth reading experience, conducive to developing focus and critical thinking skills (33, 34). On the other hand, negative news coverage may increase readers’ anxiety and depressive symptoms (35, 36).

Studies have produced contradictory findings regarding the relationship between print media consumption and mental health. Some research links traditional media use with higher levels of anxiety and depression (37, 38), potentially due to exposure to negative news and the passive nature of print media consumption (39, 40). However, other studies highlight the positive role of print media in delivering structured information and encouraging deep reflection (41, 42).

### 1.4 The mediating role of health literacy

Health literacy (HL) refers to an individual’s ability to effectively access, comprehend, evaluate, and use health information, and has been widely recognized as a key determinant of both psychological and physical health (43, 44). Early research predominantly emphasized “functional” skills such as reading and numeracy. Subsequently, Nutbeam expanded the model to include “interactive” and “critical” dimensions, highlighting the importance of communication, information appraisal, and autonomous decision-making (45). With the advent of the digital age, health literacy has evolved into electronic health literacy (eHL), which integrates multiple literacies—including traditional, information, scientific, media, and digital literacies—enabling individuals to navigate safely in a dynamic and often underregulated online environment.

As theoretical frameworks have expanded, assessment tools have also diversified. The TOFHLA (Test of Functional Health Literacy in Adults) and REALM (Rapid Estimate of Adult Literacy in Medicine) employ performance-based assessments to evaluate functional reading and numeracy but do not capture interactive or critical dimensions (46). The NVS (Newest Vital Sign) provides rapid screening of basic interpretive skills using nutrition labels, though it assesses a limited range of health literacy dimensions (47). The HLS-EU-Q47 (European Health Literacy Survey Questionnaire-47) offers broad coverage across three core domains, but its item volume can impose respondent burden (48). The HLQ (Health Literacy Questionnaire) presents individual strengths and weaknesses across nine subscales, yet lacks a unified total score (49). The eHEALS (eHealth Literacy Scale) targets self-efficacy in accessing online health information, while overlooking critical reading in print-based contexts (50).

Striking a balance between content breadth and respondent burden, the HLS-SF12 (12-item Health Literacy Survey Short Form) offers a psychometrically sound and time-efficient tool. It assesses the three major domains—healthcare, disease prevention, and health promotion—through only 12 items and can be completed in under 5 mins. The items span both digital and print-based contexts, addressing tasks such as “seeking mental health information” and “understanding health advice from the internet and newspapers,” as well as tasks requiring deeper cognitive processing, such as “weighing the pros and cons of treatment options” and “understanding medication leaflets.” The HLS-SF12 captures both online search and verification capabilities, and critical reading and emotional regulation within traditional media settings. It thus provides a robust foundation for investigating the relationships between media, HL, and mental health (51), with prior studies showing a strong bidirectional correlation between the HLS-SF12 and eHEALS (52).

Empirical evidence consistently indicates that higher health literacy alleviates symptoms of depression and anxiety by facilitating more accurate interpretation and proactive utilization of health information (53–56). However, the mediating effect may differ in direction and magnitude depending on media type. On interactive digital platforms, rapid access and evaluation of information reliability are often rewarded; in such contexts, health literacy enhances outcomes by improving source discernment and promoting help-seeking behaviors (57, 58). In contrast, print media relies on sustained reading and critical reflection; here, health literacy mitigates the emotional impact of sensational or negative content by fostering deeper understanding and balanced evaluation (59, 60). Whether these effects are primarily driven by medical knowledge, preventive orientation, or motivational factors in health promotion remains to be elucidated.

### 1.5 Age stratification and group heterogeneity

Age, as a key demographic variable, exhibits marked group differences in media use patterns, health literacy levels, and mental health conditions (61, 62). Different age cohorts display varying media preferences, technological acceptance, and information processing capacities, which may contribute to heterogeneous media effects on mental health (63, 64).

Young adults (aged 19–40) tend to favor digital interactive media and possess higher digital literacy and technological acceptance (65, 66). Middle-aged individuals (aged 41–60) demonstrate more balanced media use, engaging with both traditional and digital platforms (67, 68). Older adults (aged 61 and above) are more reliant on traditional print media and less familiar with digital technologies (69, 70).

These age-related differences also extend to the dimensions of health literacy. Younger individuals may perform better in the “disease prevention” dimension, focusing on preventive behaviors and risk management (71, 72). In contrast, older adults may show stronger engagement with “health promotion,” emphasizing overall well-being maintenance and improvement (73, 74). Therefore, the interaction between age stratification and health literacy dimensions may be key to understanding media’s effects on mental health.

### 1.6 Knowledge gaps and research questions

Despite advances in understanding the media-mental health relationship, several important knowledge gaps remain: First, most studies focus on the effects of a single media type, lacking systematic comparisons across different media formats. Although existing research has documented the risks of digital media overuse and the negative impact of social media on mental health (75, 76), the positive mechanisms and distinctions among media types have not been fully explored. Second, the mediating role of health literacy remains underexamined, particularly the specific mechanisms through which its dimensions operate within different media pathways (77, 78). Finally, although age-related heterogeneity is theoretically recognized, empirical evidence is limited, especially regarding the interactions between age stratification and health literacy dimensions (78, 79).

Against this backdrop, this study aims to systematically compare the differentiated impact pathways of digital interactive media and traditional print media on mental health—specifically depression and anxiety symptoms measured via self-reports using the PHQ-9 and GAD-7—through the theoretical lens of health literacy. It further investigates the heterogeneity of these mechanisms across age groups. Elucidating the underlying mechanisms by which different media types influence mental health has become a key priority for researchers and policymakers alike, with significant implications for developing effective media literacy education and mental health promotion strategies.

## 2 Methods

### 2.1 Conceptual framework and research hypotheses

**Figure 1:**
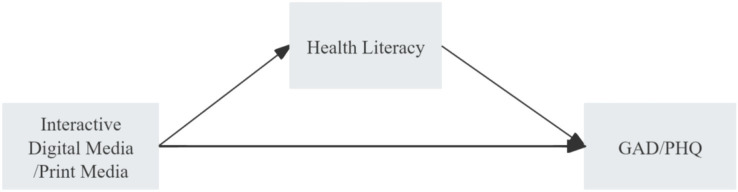
Hypothesized pathways linking digital interactive media and print media use to depression (PHQ) and anxiety (GAD).

Drawing on media use theory, health literacy theory, and life course theory, this study constructs an integrated conceptual framework to investigate the mechanisms by which print and digital interactive media affect mental health (80–82). In this framework, the frequency of print and digital media use are the independent variables; mental health status—measured by depressive symptoms (PHQ-9) and anxiety symptoms (GAD-7)—is the dependent variable; health literacy—operationalized through three dimensions: healthcare, disease prevention, and health promotion—acts as the mediating variable; and age stratification (19–40, 41–60, and 61 + years) functions as the moderating variable (Figure 1).

The conceptual framework hypothesizes that different types of media influence mental health through distinct dimensions of health literacy and that these effects are heterogeneous across age groups. It includes three main pathways: (1) a direct effects pathway, positing that print and digital media use directly impact mental health, though the direction and strength may differ; (2) a mediation pathway, proposing that media use indirectly influences mental health via the three dimensions of health literacy, with different media types operating primarily through distinct health literacy domains; and (3) a moderation pathway, suggesting that age stratification moderates the effects of media use on health literacy and mental health, leading to varied impact patterns across different age cohorts.

### 2.2 Sampling procedures

#### 2.2.1 Data source and sample

This study utilized data from the 2021 Psychology and Behavior Investigation of Chinese Residents (PBICR) (83). PBICR is a large-scale, nationally representative survey designed to regularly collect high-quality microdata on the general population, specific subpopulations, and individuals with particular conditions in China. The primary aim of the survey is to support interdisciplinary research on lifelong health by examining key determinants of psychological and behavioral outcomes among Chinese residents. The 2021 PBICR covered 34 provinces and 120 cities across China, ensuring strong regional and demographic representativeness.

A stratified multistage random sampling strategy was employed. First, community sampling quotas for each province (i.e., primary administrative unit) were determined based on population size. Then, a 3:2 ratio was used to allocate samples between urban and rural areas. Ultimately, each province contributed 10 to 60 sampled communities. Data collection was conducted via one-on-one, face-to-face interviews by trained and certified field interviewers to ensure reliability and representativeness.

PBICR inclusion criteria were as follows: individuals aged 12 and above, capable of understanding the survey content (independently or with assistance), without severe cognitive impairments, and who provided informed consent. The survey collected data on sociodemographic characteristics, family background, health behaviors, health literacy, disease status, and physical and mental health. A total of 11,031 valid responses were collected. For the purpose of this study, the analytic sample was restricted to adults aged 19 and above. After filtering based on age, 9,966 adult respondents were retained for statistical analysis. The sample represented a wide range of regions, age groups, and genders across China, thereby supporting the generalizability of the findings.

#### 2.2.2 Ethical considerations

This study conducted secondary data analysis using publicly available PBICR data. The original data collection received ethical approval from the relevant institutional review board (JKWH-2021-01), and all participants provided informed consent at the time of the original survey.

As secondary data users, the authors strictly adhered to the PBICR data usage agreement, committing to academic use only. All data analyses were anonymized, with no personally identifiable information involved. Findings are reported in aggregate statistical form. As no new human subject data collection or experimentation was conducted, additional ethical approval was not required.

### 2.3 Data collection

#### 2.3.1 Demographic characteristics

Demographic variables collected in this study included: gender (male, female); age (19–40 years, 41–60 years, 61 years and above); ethnicity (Han, ethnic minority); household registration status (agricultural, non-agricultural); residential location (rural, urban); education level (junior high school or below, high school/technical secondary school, college/bachelor’s degree, master’s/doctoral degree); marital status (single, married); employment status (student, employed, retired, unemployed/irregular work); income level (≤3,000 RMB, 3,001–7,500 RMB, ≥7,500 RMB); body mass index (BMI: underweight, normal, overweight/obese); presence of chronic disease (yes, no); and health behaviors including smoking (yes, no) and alcohol consumption (yes, no).

#### 2.3.2 Digital interactive media and print media use

Media usage was assessed using a self-developed Media Use Behavior Scale, which included two subdimensions: print media (e.g., newspapers, magazines) and digital interactive media (e.g., smartphones, computers). Each item was rated on a five-point Likert scale ranging from 1 (never) to 5 (almost daily), with higher scores indicating more frequent media use.

#### 2.3.3 Health literacy

Health literacy was measured using the Chinese version of the short-form Health Literacy Scale (HLS-SF12) developed by Tuyen V. Duong and colleagues (48, 51). This instrument evaluates individuals’ ability to access, understand, appraise, and apply health-related information across three key domains: health promotion, healthcare, and disease prevention. The scale comprises 12 items, each domain covering four cognitive processing stages. Responses were rated on a four-point Likert scale (1 = very difficult, 2 = difficult, 3 = easy, 4 = very easy), with higher scores reflecting higher health literacy levels.

The original Chinese version of the HLS-SF12 demonstrated strong reliability, with an overall Cronbach’s alpha of 0.94 and subscale reliabilities ranging from 0.86 to 0.87. In the present study, the scale showed excellent internal consistency: overall Cronbach’s alpha was 0.916; 0.905 for the healthcare domain, 0.861 for disease prevention, and 0.871 for health promotion, supporting the tool’s reliability and validity in this sample.

#### 2.3.4 Depression

Depression symptoms were assessed using the Patient Health Questionnaire-9 (PHQ-9) (84). This nine-item scale evaluates the frequency of depressive symptoms over the past 2 weeks. Each item is scored from 0 (“not at all”) to 3 (“nearly every day”), yielding a total score ranging from 0 to 27, with higher scores indicating more severe depressive symptoms.

PHQ-9 has been widely validated and is known for its high internal consistency, sensitivity, and specificity across diverse populations. In this study, the scale demonstrated excellent reliability with a Cronbach’s alpha of 0.938.

#### 2.3.5 Anxiety

Anxiety symptoms were assessed using the Generalized Anxiety Disorder-7 (GAD-7) scale (85), a widely used screening tool for generalized anxiety. The instrument comprises seven items, each scored on a four-point scale (0 = “not at all,” 3 = “nearly every day”), with total scores ranging from 0 to 21. Higher scores indicate more severe anxiety symptoms. GAD-7 has demonstrated strong reliability and validity across international settings. In the present study, the scale exhibited excellent internal consistency, with a Cronbach’s alpha of 0.955.

### 2.4 Statistical analysis

Descriptive statistics were first conducted to characterize the sample’s demographic profile and to examine the distribution of depression and anxiety symptoms across subgroups. Print media and digital interactive media use frequencies were treated as independent variables, while depression and anxiety scores served as dependent variables. The three core dimensions of health literacy were included as mediating variables.

Spearman correlation analysis was used to explore associations among the study variables. Analysis of variance (ANOVA) was applied to test differences in media use, health literacy, and mental health outcomes across age groups. To examine the significance of mediation effects, the Bootstrap method with 1,000 resampling iterations was employed.

All statistical models were adjusted for 13 potential confounding variables, including gender and age. Data analyses were performed using SPSS version 27 and PROCESS macro version 4.2. Statistical significance was defined as *p* < 0.05.

## 3 Results

### 3.1 Sample characteristics

As shown in Table 1, regarding demographic characteristics, 4,591 participants (46.07%) were male and 5,375 (53.93%) were female. A majority of the sample (53.50%, *n* = 5,332) were aged between 19 and 40 years. Most participants identified as Han ethnicity (94.33%, *n* = 9,401), held non-agricultural household registration (58.18%, *n* = 5,798), and resided in urban areas (72.80%, *n* = 7,255).

**Table 1:** Participant characteristics (*N* = 9,966).

Variable	*n* (%)
Sex	
Female	5,375 (53.93)
Male	4,591 (46.07)
Age group	
19–40 years	5,332 (53.5)
41–60 years	3,487 (34.99)
61 years and above	1,147 (11.51)
Ethnicity	
Han Chinese	9,401 (94.33)
Ethnic minority	565 (5.67)
Household registration	
Non-agricultural	5,798 (58.18)
Agricultural	4,168 (41.82)
Place of residence	
Urban	7,255 (72.8)
Rural	2,711 (27.2)
Educational attainment	
Junior high school or below	2,239 (22.47)
High school/technical school	1,598 (16.03)
College/bachelor’s degree	5,401 (54.19)
Master’s/doctoral degree	728 (7.3)
Marital status	
Married	6,226 (62.47)
Single (never married/divorced/widowed)	3,740 (37.53)
Employment status	
Employed	4,625 (46.41)
Student	2,271 (22.79)
Unemployed/irregular work	2,186 (21.93)
Retired	884 (8.87)
Monthly income (RMB)	
≦3,000	5,010 (50.27)
3,001–7,500	2,702 (27.11)
≥7,500	2,254 (22.62)
Body mass index (BMI)	
Normal	5,988 (60.08)
Overweight/Obese	2,640 (26.49)
Underweight	1,338 (13.43)
Chronic disease	
No	7,962 (79.89)
Yes	2004 (20.11)
Smoking	
No	8,585 (86.14)
Yes	1,381 (13.86)
Alcohol consumption	
No	5,769 (57.89)
Yes	4,197 (42.11)

In terms of socioeconomic characteristics, 62.47% (*n* = 6,226) were married. A total of 54.19% (*n* = 5,401) had attained a bachelor’s degree or higher, and 46.41% (*n* = 4,625) were currently employed. Half of the participants (50.27%, *n* = 5,010) reported a monthly income of ≤3,000 RMB.

Regarding health-related characteristics, 60.08% (*n* = 5,988) had a body mass index (BMI) within the normal range, and 20.11% (*n* = 2,004) reported having a chronic illness. Current smokers accounted for 13.86% (*n* = 1,381), and 42.11% (*n* = 4,197) reported alcohol consumption.

As presented in Table 2, for the key study variables, the mean health literacy subscale scores were as follows: healthcare (M = 12.116, SD = 2.242), disease prevention (M = 12.231, SD = 2.135), and health promotion (M = 12.300, SD = 2.148), indicating a generally high level of health literacy among participants. In contrast, digital interactive media were used more frequently (M = 4.334, SD = 1.131), whereas print media usage was comparatively low (M = 1.881, SD = 1.092).

**Table 2:** Descriptive statistics of study variables (*N* = 9,966).

Variable	Mean (SD)	*n* (%)
Health literacy		
Healthcare	12.116 (2.242)	
Disease prevention	12.231 (2.135)	
Health promotion	12.3 (2.148)	
Media use		
Digital interactive media	4.334 (1.131)	
Print media	1.881 (1.092)	
Mental health outcomes		
GAD-7 Score	4.47 (4.661)	
PHQ-9 Score	6.186 (5.69)	
GAD-7 (Categorical classification)		
None		5,540 (55.59)
Mild		3,059 (30.69)
Moderate		1,096 (11.00)
Severe		271 (2.72)
PHQ-9 (Categorical classification)		
None		4,543 (45.58)
Mild		3,441 (34.53)
Moderate		1,025 (10.28)
Moderately severe		732 (7.34)
Severe		225 (2.26)

GAD-7 = Generalized Anxiety Disorder 7-item scale (range: 0–21). Cut-off points: None (0–4), Mild (5–9), Moderate (10–14), Severe (15–21). PHQ-9 = Patient Health Questionnaire-9 (range: 0–27). Cut-off points: None (0–4), Mild (5–9), Moderate (10–14), Moderately Severe (15–19), Severe (20–27).

With respect to mental health outcomes, the mean scores for Generalized Anxiety Disorder (GAD-7) and depression (PHQ-9) were 4.47 (SD = 4.661) and 6.186 (SD = 5.690), respectively. Based on diagnostic thresholds, 44.41% of participants exhibited at least mild anxiety symptoms, while 54.42% reported at least mild depressive symptoms.

### 3.2 Correlation analysis

As shown in Table 3, the correlation analysis revealed significant associations among anxiety, depression, health literacy, and media use variables.

**Table 3:** Correlation matrix of study variables.

Variables	GAD-7	PHQ-9	Healthcare	Disease prevention	Health promotion	Digital interactive media
GAD-7	1					
PHQ-9	0.824**	1				
Healthcare	−0.186**	−0.179**	1			
Disease prevent	−0.198**	−0.200**	0.751**	1		
Health promotion	−0.190**	−0.185**	0.685**	0.744**	1	
Digital interactive media	−0.145**	−0.138**	0.270**	0.242**	0.297**	1
Print media	0.164**	0.178**	0.039**	0.037**	0.025*	−0.285**

GAD-7, Generalized Anxiety Disorder 7-item scale; PHQ-9, Patient Health Questionnaire-9; **p < 0.01.

Specifically, Generalized Anxiety Disorder (GAD-7) and depression (PHQ-9) scores were strongly positively correlated (*r* = 0.824, *p* < 0.01), indicating a high degree of symptom overlap at the individual level. The three dimensions of health literacy—healthcare, disease prevention, and health promotion—were also significantly and positively interrelated (*r* = 0.685 to 0.751, *p* < 0.01). However, all health literacy dimensions were negatively associated with GAD-7 and PHQ-9 scores (*r* = −0.179 to −0.200, *p* < 0.01), suggesting that higher health literacy is linked to better mental health outcomes.

In terms of media use, digital interactive media showed a significant positive correlation with all dimensions of health literacy (*r* = 0.242 to 0.297, *p* < 0.01), and a negative correlation with GAD-7 (*r* = −0.145, *p* < 0.01) and PHQ-9 (*r* = −0.138, *p* < 0.01). In contrast, print media use was positively correlated with GAD-7 and PHQ-9 scores (*r* = 0.164 to 0.178, *p* < 0.01), and negatively correlated with digital interactive media use (*r* = −0.285, *p* < 0.01).

### 3.3 Age group differences

As shown in Table 4 significant differences were observed across age groups in terms of depression, anxiety, media use, and health literacy (*p* < 0.01).

**Table 4:** Analysis of variance (ANOVA) for age group differences in key study variables.

Variables	Age (Mean ± SD)			*F*	*p*
	19–40 years (*n* = 5,332)	41–60 years (*n* = 3,487)	≥61 years (*n* = 1,147)		
GAD-7	4.81 ± 4.90	4.15 ± 4.35	3.85 ± 4.28	33.262	*p* < 0.001
PHQ-9	6.75 ± 6.01	5.63 ± 5.25	5.28 ± 5.12	57.839	*p* < 0.001
Digital interactive media	4.51 ± 0.91	4.46 ± 0.98	3.11 ± 1.61	901.668	*p* < 0.001
Print media	1.82 ± 1.06	1.89 ± 1.06	2.14 ± 1.29	39.287	*p* < 0.001
Healthcare	12.41 ± 2.17	12.07 ± 2.17	10.91 ± 2.37	220.571	*p* < 0.001
Disease prevent	12.44 ± 2.13	12.17 ± 2.07	11.44 ± 2.13	109.303	*p* < 0.001
Health promotion	12.58 ± 2.09	12.23 ± 2.08	11.20 ± 2.25	205.218	*p* < 0.001

GAD-7, Generalized Anxiety Disorder 7-item scale; PHQ-9, Patient Health Questionnaire-9.

Young adults aged 19–40 years reported significantly higher levels of anxiety and depression symptoms, with mean scores of 4.81 (SD = 4.90) for GAD-7 and 6.75 (SD = 6.01) for PHQ-9. These scores were notably higher than those of middle-aged participants aged 41–60 years (GAD-7: 4.15 ± 4.35; PHQ-9: 5.63 ± 5.25) and older adults aged 61 years and above (GAD-7: 3.85 ± 4.28; PHQ-9: 5.28 ± 5.12).

Regarding media use, the frequency of digital interactive media use showed a clear declining trend with increasing age. Participants aged 19–40 (M = 4.51, SD = 0.91) and 41–60 (M = 4.46, SD = 0.98) reported significantly higher usage compared to those aged 61 and older (M = 3.11, SD = 1.61). In contrast, print media use exhibited a positive correlation with age: older adults reported the highest usage (M = 2.14, SD = 1.29), significantly exceeding the younger groups.

For all three dimensions of health literacy—healthcare, disease prevention, and health promotion—a gradual decline in scores was observed with advancing age. The 19–40 age group scored the highest in healthcare (M = 12.41, SD = 2.17), disease prevention (M = 12.44, SD = 2.13), and health promotion (M = 12.58, SD = 2.09), while the oldest group recorded the lowest scores across all domains.

### 3.4 Mediating role of health literacy

**Figure 2:**
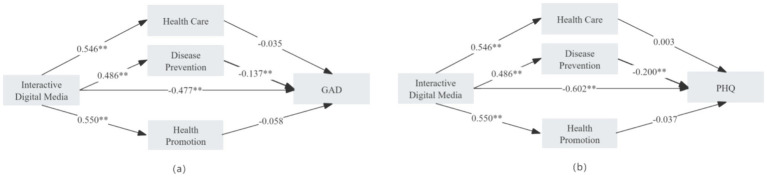
Mediating pathways of health literacy dimensions in the relationship between digital interactive media use and GAD/PHQ.

As illustrated in Figure 2 and detailed in Appendices 1, 2, the inclusion of health literacy as a mediating variable revealed that digital interactive media continued to exert significant direct effects on both GAD-7 and PHQ-9 scores (GAD: *β* = −0.477, 95% CI [−0.567, −0.388], *p* < 0.001; PHQ: *β* = −0.602, 95% CI [−0.710, −0.493], *p* < 0.001).

Meanwhile, the mediation analysis demonstrated that health literacy served as a partial mediator in the relationship between digital interactive media use and both anxiety and depression. Specifically, the “disease prevention” dimension of health literacy mediated the effect of digital media on GAD-7 with an effect size of −0.067 (95% CI [−0.105, −0.025]), and on PHQ-9 with an effect size of −0.097 (95% CI [−0.145, −0.049]), both of which were statistically significant. These findings support the role of health literacy—particularly the disease prevention dimension—as a critical bridge linking digital interactive media use with improved mental health outcomes.

Further path analysis revealed that digital interactive media use significantly positively predicted all three dimensions of health literacy: healthcare (*β* = 0.546, 95% CI [0.507, 0.585], *p* < 0.001), disease prevention (*β* = 0.486, 95% CI [0.448, 0.524], *p* < 0.001), and health promotion (*β* = 0.550, 95% CI [0.512, 0.587], *p* < 0.001). However, among these, only the “disease prevention” dimension significantly negatively predicted GAD-7 (*β* = −0.137, 95% CI [−0.220, −0.054], *p* < 0.001) and PHQ-9 (*β* = −0.200, 95% CI [−0.301, −0.099], *p* < 0.001). The other two dimensions did not exhibit statistically significant mediating effects.

**Figure 3:**
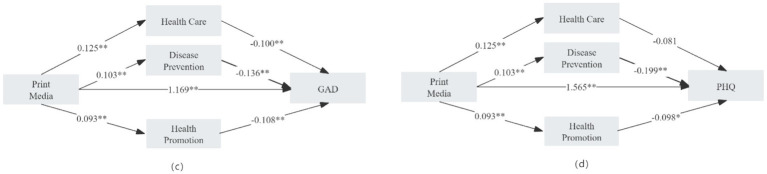
Mediating pathways of health literacy dimensions in the relationship between print media use and GAD/PHQ.

As shown in Appendices 3, 4 and Figure 3, the path analysis for print media use revealed that all three dimensions of health literacy—disease prevention, healthcare, and health promotion—exhibited a suppressing effect on the associations between print media use and both GAD-7 and PHQ-9 outcomes.

Specifically, in the GAD-7 model, the total effect of print media use was 1.133 (95% CI [1.051, 1.214], *p* < 0.001). After including health literacy as a mediator, the direct effect increased to 1.169 (95% CI [1.089, 1.249], *p* < 0.001).

Similarly, in the PHQ-9 model, the total effect was *β* = 1.525 (95% CI [1.427, 1.623], *p* < 0.001), and the direct effect increased to *β* = 1.565 (95% CI [1.468, 1.662], *p* < 0.001) upon adjusting for health literacy.

### 3.5 Age-based heterogeneity in the mediating role of health literacy

To further explore whether the mediating role of health literacy in the relationship between media exposure and mental health varies by age, separate mediation analyses were conducted for three age groups: 19–40 years, 41–60 years, and 61 years and above (see Appendices 5–7). The results revealed clear heterogeneity across age groups, as detailed below:

In the 19–40 age group, digital interactive media exerted a partial mediating effect on PHQ-9 through the “disease prevention” dimension (*β* = −0.114, 95% CI [−0.029, −0.003], *p* < 0.01). Print media use demonstrated a suppressing effect through the same dimension on both GAD-7 (*β* = −0.009, 95% CI [−0.006, −0.000], *p* < 0.01) and PHQ-9 (*β* = −0.015, 95% CI [−0.007, −0.000], *p* < 0.01).

In the 41–60 age group, digital interactive media also showed partial mediation effects through “disease prevention” on both GAD-7 (*β* = −0.069, 95% CI [−0.031, −0.002], *p* < 0.01) and PHQ-9 (*β* = −0.083, 95% CI [−0.031, −0.003], *p* < 0.01). Print media demonstrated suppressing effects on both outcomes through “disease prevention” (GAD-7: *β* = −0.021, 95% CI [−0.012, −0.001], *p* < 0.01; PHQ-9: *β* = −0.025, 95% CI [−0.012, −0.001], *p* < 0.01), and also showed a significant effect on GAD-7 via the “health promotion” dimension (*β* = −0.016, 95% CI [−0.010, −0.000], *p* < 0.01).

In the 61 and above age group, digital interactive media had a fully mediating effect on both GAD-7 (*β* = −0.111, 95% CI [−0.070, −0.010], *p* < 0.01) and PHQ-9 (*β* = −0.116, 95% CI [−0.066, −0.011], *p* < 0.01) through the “health promotion” dimension. Conversely, print media use demonstrated suppressing effects on GAD-7 (*β* = −0.046, 95% CI [−0.028, −0.004], *p* < 0.01) and PHQ-9 (*β* = −0.048, 95% CI [−0.026, −0.003], *p* < 0.01) via the same dimension.

## 4 Discussion

This study explored the psychological mechanisms through which digital interactive media and print media influence anxiety and depression, with a particular focus on the mediating role of health literacy and its heterogeneity across age groups. The findings not only confirmed the direct effects of media use on mental health but also revealed the complex and differentiated mediating pathways of health literacy, offering valuable insights for both theoretical refinement and practical intervention within the Chinese cultural context.

First, this study explicitly acknowledges the distinction between digital interactive media and print media, emphasizing that digital media functions within an online environment characterized by interactivity, immediacy, and customization, whereas print media operates within a static and delayed information environment. This ecological difference is critical for understanding the differentiated psychological outcomes associated with each media type. The results confirmed significant differences between digital interactive media and print media in predicting anxiety and depression, supporting the hypothesis that different media types have distinct pathways of impact on mental health. Specifically, the use of digital interactive media was negatively associated with anxiety and depression scores, diverging from previous findings primarily based on adolescent samples (86–88). This suggests that in Chinese adult populations, the mental health outcomes of media use are influenced by user characteristics, usage patterns, and motivations. This finding is particularly relevant in a society like China, where digital media penetration is extremely high, with over 1 billion internet users and widespread adoption of social platforms such as WeChat and Weibo (89). The protective role of digital interactive media may be attributed to its functions in facilitating social connections and community support, which are especially significant in a collectivist culture like China (90). Furthermore, the results imply a potential nonlinear relationship between media use and mental health—moderate, instrumental use of digital media may offer protective effects, whereas excessive or disordered use could be detrimental (34).

In contrast, exposure to print media was positively associated with symptoms of anxiety and depression, challenging the traditional assumption of print media’s positive role in health communication (39, 88). In the digital era, the static nature, temporal delay, and tendency for negative narratives in print media may lead psychologically vulnerable individuals to repeatedly encounter adverse information, exacerbating rumination and emotional distress (91, 92). This phenomenon is particularly evident in the Chinese media landscape, where traditional newspapers often emphasize crisis events and health risks, potentially creating a “cultivation effect”—a process where prolonged exposure to such content heightens perceptions of health threats and personal vulnerability (91). Moreover, the perceived authority of print media in China’s media consumption hierarchy may amplify the psychological effects of negative health information (92). Among high-risk populations with low digital literacy and limited ability to access balanced health information, this negative impact may be more pronounced (93). These findings suggest that mental health interventions should not only enhance individual health literacy but also optimize the media structure at a systemic level—for example, by strengthening the social support functions of digital media and addressing sedentary behavior risks associated with print media use.

Second, the study confirmed the mediating role of health literacy in the relationship between media use and mental health, validating the hypothesis that media influence mental health through indirect pathways. While both media types can enhance health literacy, digital interactive media exhibited a more pronounced effect (94). Its primary mechanism involves improving disease prevention capabilities, thereby alleviating anxiety and depression symptoms. This may be due to the interactivity and accessibility of digital media, which allows users to perceive greater control and information certainty during health information acquisition, thereby reducing anxiety (95, 96). In a society that traditionally emphasizes disease prevention, such as China, this result aligns well with cultural values and resonates with the preventive focus of traditional Chinese medicine. The interactive and customizable nature of digital platforms enables users to engage with health content in ways that meet their personal needs, facilitating cultural understanding and acceptance.

By comparison, the health literacy pathway associated with print media demonstrated a “suppressing effect”: despite its overall negative association with mental health, the health information conveyed by print media did contribute to improving users’ health literacy, partially mitigating its adverse psychological effects. This indicates that the mode of dissemination and presentation style of different media may influence both the direction and strength of health literacy’s impact. In the Chinese context, this finding may reflect the lingering authority of traditional media—despite potentially anxiety-inducing content, people still place considerable trust in print sources.

To support the rationale for using HLS-SF12 in this context, it is important to note that previous studies have validated its applicability in Chinese populations across diverse age groups and health conditions (48, 51). These studies demonstrated the tool’s sensitivity in detecting differences in health literacy levels related to both digital and offline health behaviors. Therefore, our selection of HLS-SF12 is grounded in its multidimensional structure, time efficiency, and proven applicability in large-scale public health research. Additionally, prior studies have also found a strong bidirectional association between HLS-SF12 and eHEALS (52). The identification of these complex and differentiated mediating pathways underscores the importance of the methodological approach employed in health literacy measurement. The choice of the HLS-SF12 over specialized digital health literacy scales (such as eHEALS) was strategically aligned with the study’s comparative framework. The HLS-SF12’s multi-dimensional structure across healthcare, disease prevention, and health promotion domains enabled the detection of nuanced mediating mechanisms that would have been obscured by single-dimension measures. Specifically, the scale’s inclusion of items such as “understanding media information about becoming healthier” provided a unified measurement standard for comparing different media types, while items like “finding information on how to manage mental health problems” directly corresponded to the outcome variables. This methodological design proved essential for revealing that digital media primarily operates through the “disease prevention” dimension, whereas print media exhibits suppressing effects across all three dimensions—a finding that validates the necessity of using comprehensive, multi-dimensional health literacy assessments in media effects research. Furthermore, the scale’s cross-cultural validation in Chinese populations and its effectiveness across different age groups (particularly relevant given that 46.5% of the participants were aged 41 and above) ensured measurement equivalence across the diverse sample, thereby strengthening the reliability of the comparative analyses.

Third, in light of the heterogeneous definitions and conceptualizations of health literacy across the literature, we acknowledge the potential limitations of our operational approach. While the HLS-SF12 effectively captures general health literacy across multiple domains, it may underrepresent certain competencies specific to the digital context—such as algorithmic discernment or platform navigation—that are more precisely measured by digital health literacy tools like eHEALS. This underscores the need for future research to develop integrative tools that can holistically evaluate media-specific health literacy in increasingly hybrid information environments. The study also identified significant age-related heterogeneity in the mediating role of health literacy between media use and mental health, supporting the hypothesis of differentiated pathways across age groups. Among young and middle-aged adults, the “disease prevention” dimension played a key mediating role in the relationship between media exposure and mental health, likely due to their greater initiative in seeking and applying preventive health information (64, 97). In contrast, among older adults (aged 61 and above), the “health promotion” dimension emerged as the primary mediator, indicating a preference for specific guidance on daily behavior management to enhance psychological well-being (98, 99). This intergenerational difference may reflect varying health beliefs across generations in Chinese society: younger individuals tend to favor proactive health management, while older adults prefer maintaining health through traditional routines. This finding highlights the need for age-targeted intervention strategies—for instance, promoting prevention-oriented health education among the young, and designing cognitively friendly, behaviorally explicit communication schemes for the older adult(s).

In addition, attention should be paid to the culturally specific context of mental health in China. Despite growing public awareness, mental health stigma remains pervasive, with many individuals reluctant to seek professional help due to concerns about social judgment and family honor. This cultural backdrop may shape how individuals process health information and may also explain why the anonymity and accessibility of digital media can offer psychological protection that traditional print media cannot.

## 5 Strengths and limitations

This study has several significant strengths. First, it is among the first to systematically compare the mental health impacts of digital interactive and print media within the Chinese cultural context, filling a theoretical gap in this field. Second, by incorporating health literacy as a mediating variable, the research provides an in-depth understanding of the mechanisms through which media affects mental health, which could inform the development of evidence-based mental health intervention strategies.

Of course, this study also has several limitations. First, its cross-sectional design limits causal inference; therefore, future research should employ longitudinal designs to verify the proposed pathways. Second, the study did not distinguish among specific types of digital media (e.g., social media, health-related apps), a differentiation that warrants further investigation in subsequent studies. Third, since the sample was restricted to Chinese adults, the cross-cultural generalizability of the findings remains uncertain, underscoring the need for multicultural comparative research. Fourth, the study did not provide a clear operational definition or categorization of health literacy levels (e.g., low, medium, high), which may affect the explanatory power of the mediation effects. Future research should address this issue in greater depth.

In addition, while the selection of the HLS-SF12 was grounded in comprehensive theoretical and empirical considerations, we acknowledge certain limitations associated with this choice. In the digital era, although the use of HLS-SF12 was justified by its multidimensional coverage of health literacy domains, brevity, and validated applicability in Chinese populations, and prior research has demonstrated a strong bidirectional association between HLS-SF12 and eHEALS (52), specialized digital health literacy instruments such as eHEALS may more precisely capture specific competencies for processing health information in digital environments. This suggests that future research may adopt a combined measurement approach using both tools to assess general and digital-specific health literacy, thereby enhancing predictive accuracy in media–mental health research. Due to the issues of inconsistent definitions and measurement differentiation in the current concept of health literacy across functional dimensions, media contexts, and technological evolution, the HLS-SF12, despite its comprehensiveness and generalizability, has not fully captured certain digital behaviors that heavily rely on platform operations and social algorithms, which constitutes a definite limitation. Therefore, this study calls for the future development of a composite assessment tool that combines a general structure with media-sensitive dimensions.

Fifth, the mental health variables were broadly categorized as anxiety and depression, without refinement into specific diagnostic criteria or stages, potentially oversimplifying the complexity of psychological issues. Sixth, the study did not detail the types and contents of print media used, particularly whether the content was tailored to different health literacy groups—an important consideration. Finally, the study did not control for potential confounding variables such as socioeconomic status, media accessibility, or cultural factors, which could influence the strength and direction of the relationship between media use and mental health.

In summary, this study not only clarifies the mediating role of health literacy but also reveals age-related heterogeneous pathways, contributing valuable insights for the theoretical foundations of media ecology, health communication, and mental health research. The findings provide practical pathways for developing culturally adaptive and age-sensitive media intervention strategies.

## 6 Conclusion

Drawing on the theoretical framework of health literacy, this study systematically compared the differential effects of digital interactive media and traditional print media on depression and anxiety symptoms, and examined the age-related heterogeneity of these associations. The results indicated that digital interactive media use was positively associated with health literacy, and not only directly reduced depression and anxiety levels but also partially mediated these effects through the “disease prevention” dimension of health literacy.

In contrast, print media use was positively associated with mental health symptoms, and all three dimensions of health literacy—healthcare, disease prevention, and health promotion—showed suppression effects on these relationships. Importantly, the mediating pathways differed significantly across age groups. These findings offer critical insights for designing precision mental health interventions that take into account age-specific cognitive characteristics and media preferences.

This study contributes to the empirical evidence on media use and mental health, and theoretically delineates how different dimensions of health literacy function within this relationship. It provides a refined analytical framework for future research integrating media typologies and health literacy mechanisms. However, given its cross-sectional nature, longitudinal and cross-contextual studies are essential to establish causality and enhance external validity. Future research should adopt interdisciplinary and large-sample longitudinal approaches to offer more robust support for mental health intervention planning and policy-making.

## Data Availability

The data analyzed in this study is subject to the following licenses/restrictions: researchers interested in accessing the data may submit a reasonable request to the PBICR research team. Requests to access these datasets should be directed to https://www.x-mol.com/groups/pbicr.
