# Supplementary material for: Differential impacts of digital interactive media and print media on mental health: mediating mechanism of health literacy and group heterogeneity
Source: Front Public Health. 2025 Sep 4;13:1618570. doi: 10.3389/fpubh.2025.1618570 (PMC12443551; doi:10.3389/fpubh.2025.1618570)
Supplement: Supplementary file 1 [file Table_1.docx]

Appendix 1. Mediation Analysis of Health Literacy in the Relationship Between Digital Interactive Media Use and GAD-7

| Path | Meaning | β | 95% CI | | SE | p |
| --- | --- | --- | --- | --- | --- | --- |
|  |  |  | LLCI | ULCI |  |  |
| Digital Interactive Media=>Health Care=>GAD | Indirect Effect | -0.019 | -0.056 | 0.017 | 0.019 | *p*<0.001 |
| Digital Interactive Media=>Health Care | X=>M | 0.546 | 0.507 | 0.585 | 0.02 | *p*<0.001 |
| Health Care=>GAD | M=>Y | -0.034 | -0.105 | 0.036 | 0.036 | 0.336 |
| Digital Interactive Media=>GAD | Direct Effect | -0.477 | -0.567 | -0.388 | 0.046 | *p*<0.001 |
| Digital Interactive Media=>GAD | Total Effect | -0.595 | -0.68 | -0.509 | 0.044 | *p*<0.001 |
| Digital Interactive Media=>Disease Prevention=>GAD | Indirect Effect | -0.067 | -0.105 | -0.025 | 0.021 | *p*<0.001 |
| Digital Interactive Media=>Disease Prevention | X=>M | 0.486 | 0.448 | 0.524 | 0.019 | *p*<0.001 |
| Disease Prevention=>GAD | M=>Y | -0.137 | -0.22 | -0.054 | 0.042 | 0.001 |
| Digital Interactive Media=>GAD | Direct Effect | -0.477 | -0.567 | -0.388 | 0.046 | *p*<0.001 |
| Digital Interactive Media=>GAD | Total Effect | -0.595 | -0.68 | -0.509 | 0.044 | *p*<0.001 |
| Digital Interactive Media=>Health Promotion=>GAD | Indirect Effect | -0.032 | -0.074 | 0.012 | 0.022 | *p*<0.001 |
| Digital Interactive Media=>Health Promotion | X=>M | 0.55 | 0.512 | 0.587 | 0.019 | *p*<0.001 |
| Health Promotion=>GAD | M=>Y | -0.058 | -0.133 | 0.016 | 0.038 | 0.125 |
| Digital Interactive Media=>GAD | Direct Effect | -0.477 | -0.567 | -0.388 | 0.046 | *p*<0.001 |
| Digital Interactive Media=>GAD | Total Effect | -0.595 | -0.68 | -0.509 | 0.044 | *p*<0.001 |

Note:GAD = Generalized Anxiety Disorder 7-item scale；PHQ = Patient Health Questionnaire-9

Appendix 2. Mediation Analysis of Health Literacy in the Relationship Between Digital Interactive Media Use and PHQ-9

| Path | Meaning | β | 95% CI | | SE | p |
| --- | --- | --- | --- | --- | --- | --- |
|  |  |  | LLCI | ULCI |  |  |
| Digital Interactive Media=>Health Care=>PHQ | Indirect Effect | 0.002 | -0.043 | 0.047 | 0.023 | 0.676 |
| Digital Interactive Media=>Health Care | X=>M | 0.546 | 0.507 | 0.585 | 0.020 | *p*<0.001 |
| Health Care=>PHQ | M=>Y | 0.003 | -0.082 | 0.089 | 0.044 | 0.942 |
| Digital Interactive Media=>PHQ | Direct Effect | -0.602 | -0.71 | -0.493 | 0.055 | *p*<0.001 |
| Digital Interactive Media=>PHQ | Total Effect | -0.717 | -0.821 | -0.613 | 0.053 | *p*<0.001 |
| Digital Interactive Media=>Disease Prevention=>PHQ | Indirect Effect | -0.097 | -0.145 | -0.049 | 0.025 | *p*<0.001 |
| Digital Interactive Media=>Disease Prevention | X=>M | 0.486 | 0.448 | 0.524 | 0.019 | *p*<0.001 |
| Disease Prevention=>PHQ | M=>Y | -0.2 | -0.301 | -0.099 | 0.052 | *p*<0.001 |
| Digital Interactive Media=>PHQ | Direct Effect | -0.602 | -0.71 | -0.493 | 0.055 | *p*<0.001 |
| Digital Interactive Media=>PHQ | Total Effect | -0.717 | -0.821 | -0.613 | 0.053 | *p*<0.001 |
| Digital Interactive Media=>Health Promotion=>PHQ | Indirect Effect | -0.02 | -0.069 | 0.030 | 0.026 | *p*<0.001 |
| Digital Interactive Media=>Health Promotion | X=>M | 0.55 | 0.513 | 0.587 | 0.019 | *p*<0.001 |
| Health Promotion=>PHQ | M=>Y | -0.037 | -0.127 | 0.054 | 0.046 | 0.424 |
| Digital Interactive Media=>PHQ | Direct Effect | -0.602 | -0.71 | -0.493 | 0.055 | *p*<0.001 |
| Digital Interactive Media=>PHQ | Total Effect | -0.717 | -0.821 | -0.613 | 0.053 | *p*<0.001 |

Note:GAD = Generalized Anxiety Disorder 7-item scale；PHQ = Patient Health Questionnaire-9

Appendix 3. Mediation Analysis of Health Literacy in the Relationship Between Print Media Use and GAD-7

| Path | Meaning | β | 95% CI | | SE | p |
| --- | --- | --- | --- | --- | --- | --- |
|  |  |  | LLCI | ULCI |  |  |
| Print Media=>Health Care=>GAD | Indirect Effect | -0.012 | -0.022 | -0.004 | 0.005 | *p*<0.001 |
| Print Media=>Health Care | X=>M | 0.125 | 0.086 | 0.164 | 0.02 | *p*<0.001 |
| Health Care=>GAD | M=>Y | -0.1 | -0.167 | -0.032 | 0.035 | 0.004 |
| Print Media=>GAD | Direct Effect | 1.169 | 1.089 | 1.249 | 0.041 | *p*<0.001 |
| Print Media=>GAD | Total Effect | 1.133 | 1.051 | 1.214 | 0.041 | *p*<0.001 |
| Print Media=>Disease Prevention=>GAD | Indirect Effect | -0.014 | -0.026 | -0.005 | 0.005 | *p*<0.001 |
| Print Media=>Disease Prevention | X=>M | 0.103 | 0.065 | 0.14 | 0.019 | *p*<0.001 |
| Disease Prevention=>GAD | M=>Y | -0.136 | -0.217 | -0.056 | 0.041 | 0.001 |
| Print Media=>GAD | Direct Effect | 1.169 | 1.089 | 1.249 | 0.041 | *p*<0.001 |
| Print Media=>GAD | Total Effect | 1.133 | 1.051 | 1.214 | 0.041 | *p*<0.001 |
| Print Media=>Health Promotion=>GAD | Indirect Effect | -0.01 | -0.019 | -0.003 | 0.004 | *p*<0.001 |
| Print Media=>Health Promotion | X=>M | 0.093 | 0.056 | 0.131 | 0.019 | *p*<0.001 |
| Health Promotion=>GAD | M=>Y | -0.108 | -0.179 | -0.037 | 0.036 | 0.003 |
| Print Media=>GAD | Direct Effect | 1.169 | 1.089 | 1.249 | 0.041 | *p*<0.001 |
| Print Media=>GAD | Total Effect | 1.133 | 1.051 | 1.214 | 0.041 | *p*<0.001 |

Note:GAD = Generalized Anxiety Disorder 7-item scale；PHQ = Patient Health Questionnaire-9

Appendix 4. Mediation Analysis of Health Literacy in the Relationship Between Print Media Use and PHQ-9

| Path | Meaning | β | 95% CI | | SE | p |
| --- | --- | --- | --- | --- | --- | --- |
|  |  |  | LLCI | ULCI |  |  |
| Print Media=>Health Care=>PHQ | Indirect Effect | -0.01 | -0.022 | -0.001 | 0.005 | *p*<0.001 |
| Print Media=>Health Care | X=>M | 0.125 | 0.086 | 0.164 | 0.02 | *p*<0.001 |
| Health Care=>PHQ | M=>Y | -0.081 | -0.163 | 0 | 0.042 | 0.051 |
| Print Media=>PHQ | Direct Effect | 1.565 | 1.468 | 1.662 | 0.049 | *p*<0.001 |
| Print Media=>PHQ | Total Effect | 1.525 | 1.427 | 1.622 | 0.05 | *p*<0.001 |
| Print Media=>Disease Prevention=>PHQ | Indirect Effect | -0.02 | -0.035 | -0.009 | 0.006 | *p*<0.001 |
| Print Media=>Disease Prevention | X=>M | 0.103 | 0.065 | 0.14 | 0.019 | *p*<0.001 |
| Disease Prevention=>PHQ | M=>Y | -0.199 | -0.295 | -0.102 | 0.049 | *p*<0.001 |
| Print Media=>PHQ | Direct Effect | 1.565 | 1.468 | 1.662 | 0.049 | *p*<0.001 |
| Print Media=>PHQ | Total Effect | 1.525 | 1.427 | 1.622 | 0.05 | *p*<0.001 |
| Print Media=>Health Promotion=>PHQ | Indirect Effect | -0.009 | -0.019 | -0.001 | 0.005 | *p*<0.001 |
| Print Media=>Health Promotion | X=>M | 0.093 | 0.057 | 0.131 | 0.019 | *p*<0.001 |
| Health Promotion=>PHQ | M=>Y | -0.1 | -0.186 | -0.014 | 0.044 | 0.023 |
| Print Media=>PHQ | Direct Effect | 1.565 | 1.468 | 1.662 | 0.049 | *p*<0.001 |
| Print Media=>PHQ | Total Effect | 1.525 | 1.427 | 1.623 | 0.05 | *p*<0.001 |

Note:GAD = Generalized Anxiety Disorder 7-item scale；PHQ = Patient Health Questionnaire-9
